# *Cdkn1c *(*p57*^*Kip2*^) is the major regulator of embryonic growth within its imprinted domain on mouse distal chromosome 7

**DOI:** 10.1186/1471-213X-7-53

**Published:** 2007-05-21

**Authors:** Stuart C Andrews, Michelle D Wood, Simon J Tunster, Sheila C Barton, M Azim Surani, Rosalind M John

**Affiliations:** 1 Cardiff School of Biosciences, Cardiff University, Cardiff, UK; 2 The Wellcome Trust/Cancer Research UK Gurdon Institute, The Henry Wellcome Building of Cancer and Developmental Biology, University of Cambridge, Cambridge, UK

## Abstract

**Background:**

*Cdkn1c *encodes an embryonic cyclin-dependant kinase inhibitor that acts to negatively regulate cell proliferation and, in some tissues, to actively direct differentiation. This gene, which is an imprinted gene expressed only from the maternal allele, lies within a complex region on mouse distal chromosome 7, called the IC2 domain, which contains several other imprinted genes. Studies on mouse embryos suggest a key role for genomic imprinting in regulating embryonic growth and this has led to the proposal that imprinting evolved as a consequence of the mismatched contribution of parental resources in mammals.

**Results:**

In this study, we characterised the phenotype of mice carrying different copy number integrations of a bacterial artificial chromosome spanning *Cdkn1c*. Excess *Cdkn1c *resulted in embryonic growth retardation that was dosage-dependent and also responsive to the genetic background. Two-fold expression of *Cdkn1c *in a subset of tissues caused a 10–30% reduction in embryonic weight, embryonic lethality and was associated with a reduction in the expression of the potent, non-imprinted embryonic growth factor, *Igf1*. Conversely, loss of expression of *Cdkn1c *resulted in embryos that were 11% heavier with a two-fold increase in *Igf1*.

**Conclusion:**

We have shown that embryonic growth in mice is exquisitely sensitive to the precise dosage of *Cdkn1c*. *Cdkn1c *is a maternally expressed gene and our findings support the prediction of the parental conflict hypothesis that that the paternal genome silences genes that have an inhibitory role in embryonic growth. Within the IC2 imprinted domain, *Cdkn1c *encodes the major regulator of embryonic growth and we propose that *Cdkn1c *was the focal point of the selective pressure for imprinting of this domain.

## Background

Genomic imprinting is an unusual epigenetic phenomenon that results in the absence of expression of one copy of an autosomal gene. This allele-specific expression is initiated by events that take place within the male and female germ line during gametogenesis [1]. The parental alleles acquire an epigenetic signature that is recognised in the somatic cell and, as a result, there is permanent and heritable silencing of one parental allele. Studies on imprinted genes in mice suggest a general role for imprinting in the regulation of embryonic growth [2-8]. Two key genes that regulate embryonic growth, insulin-like growth factor 2 (Igf2) and insulin-like growth factor receptor 2 (M6P/Igf2r) are oppositely imprinted. In mice, insulin-like growth factor 1 (Igf1) and the imprinted Igf2 are thought to be the two major growth signalling molecules. These factors work in combination with at least three receptors, the insulin receptor (Insr), insulin-like growth factor receptor 1 (Igf1r) and the imprinted Igf2r to exert local and systemic effects on growth [3].

Single genetic or epigenetic changes can deregulate the function of an imprinted gene and they are particularly vulnerable targets for numerous human pathologies (recently reviewed in [9-11]. Since the cost of adopting monoallellic gene expression to an individual is clearly quite high, there must be a compelling reason for mammals to imprint their genes. The most widely accepted model that has been proposed to explain the evolution of genomic imprinting is known as the parental conflict hypothesis [12,13]. This hypothesis focuses on the asymmetry in mammals between parental contributions to the developing fetus. Although larger offspring are thought to be of benefit to both parents, in females there must be a balance between the fitness of the offspring and survival of the mother. Genomic imprinting of the paternal genome may have evolved to promote growth of the fetus while imprinting of the female genome occurred to counteract the effects of the paternal genome in order for the mother to survive and produce more offspring. However, deficiency in expression of an imprinted gene does not always lead to an embryonic growth phenotype [14]. Careful studies have revealed effects on mammalian behaviour for several genes [6,7,15-19] which suggests that the role of genomic imprinting may be more complex or that some genes are imprinted by virtue of their proximity to imprinting centres (ICs).

While studies on loss of function are important for our understanding of gene function by itself, over expression studies may provide more unequivocal information on the significance of imprinting of a particular gene. Biallelic gene expression can be studied in mice with uniparental disomy [20-22] or in mice where a targeted deletion of an imprinting centre (IC) has released genes from imprinting [23-25]. These studies generally involve multiple genes and, in regions where there are both paternally and maternally expressed genes, can also involve loss of expression. The primary advantage of using large genomic clones to analyse imprinted genes is that the exact nature and number of genes is defined by the transgenic sequence, there is no loss of expression and the genes are expressed from their endogenous promoters at the appropriate level and time, predominantly without being affected by the site of integration [26].

*Cdkn1c*, also known as *p57*^*Kip2*^, encodes a maternally expressed, cyclin-dependant kinase inhibitor (CDKi) [27-30]. Expression is tissue-specific and at high levels in cells entering mitotic arrest [27,28]. Loss of expression of *Cdkn1c *through maternal inheritance of a targeted deletion results in severe developmental abnormalities [31,32], similar to that seen in humans with loss of *CDKN1C *in Beckwith-Wiedemann syndrome (BWS) [33-37]. As with all CDKis, excess Cdkn1c induces cell cycle arrest [27,28]. Forced expression of *Cdkn1c *in retinal cultures leads to premature cell cycle exit [38] and *Cdkn1c *co-expression with *Nurr1 *promotes maturation of a midbrain dopaminergic neuronal cell line [39] demonstrating a role for Cdkn1c in both cell cycle regulation and cellular differentiation.

The *Cdkn1c *gene is located within a large cluster of imprinted genes on mouse distal chromosome 7 [29]. Maternal disomy of this region results in embryonic and extra embryonic growth retardation and embryonic lethality [22]. There are two ICs within this domain controlling two mechanistically distinct sub domains [25,40,41]. Imprinting of *Cdkn1c *is achieved through IC2, also known as *KvDMR1 *[25] and mice paternally inheriting a targeted deletion of the *KvDMR1 *locus show growth retardation. Growth retardation is also associated with maternal transmission of an 800 kb YAC transgene spanning eleven genes from *Nap1l4 *to *Th *but excluding the IC1 domain [42]. All these studies involve multiple imprinted genes and dissecting which gene is responsible for a particular phenotype is not straightforward. Nonetheless, the proportionality of the growth retardation in all these mice suggests a systemic effect on growth. Since *Igf2*, which encodes a potent embryonic mitogen, is unaffected by the *KvDMR1 *deletion, one or more of the other deregulated genes may play a novel role in regulating whole organism growth.

In this study, we characterised the phenotype of mice carrying different copy number integrations of a bacterial artificial chromosome spanning *Cdkn1c*. We show copy-number dependent embryonic growth retardation that is most severe on the 129/Sv genetic background. By using a modified BAC transgene spanning the same genomic region but with an inactivated *Cdkn1c*, we can assign the embryonic phenotype to *Cdkn1c *alone. These data indicate that *Cdkn1c *encodes the most important regulator of embryonic growth within the IC2 domain on mouse distal chromosome 7.

## Results

### Excess *Cdkn1c *causes embryonic lethality

**Figure 1:**
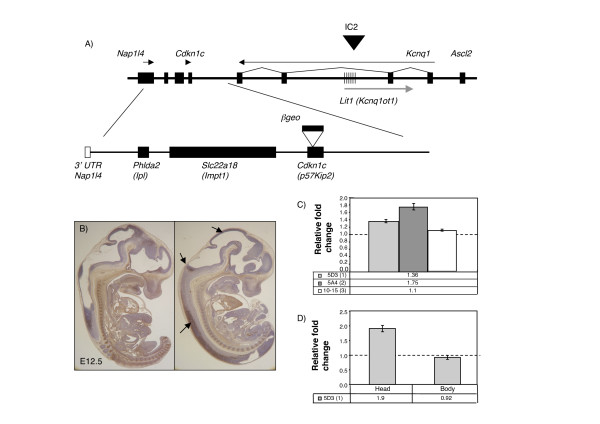
**Physical map of the telomeric end of the imprinted domain in mouse distal chromosome 7 in relation to the position of the BAC transgene and expression analysis of transgenic lines**. A) Top line shows a genomic map of the IC2 region on distal mouse chromosome 7. Hatched region marks the *KvDMR1 *region that is methylated only on the maternal allele. The black arrowhead marks the position of the IC2 control region for the domain. Arrows indicate direction of transcription. Below is the map of the 85 kb *Cdkn1c *transgene (BAC144D14). Filled boxes show the positions of the intact genes. In addition to *Phlda2*, *Slc22a18 *and *Cdkn1c*, the transgene includes the 3' UTR of *Napl14 *(white box) but not the 5' UTR. The modified BAC used to generate line 10–15 has a β-*galactosidase-neomycin *(β-*geo*) fusion gene inserted into *Cdkn1c *indicated by the white arrowhead and black filled box. B) Localisation of the Cdkn1c protein expressed from the BAC transgene. Sagittal section of E12.5 wild type and *Cdkn1c*-null/BAC transgene (KO/Tg) embryos stained with a Cdkn1c-specific antibody. Endogenous Cdkn1c is most strongly expressed in skeletal and cardiac muscle, cartilage and the developing pituitary with lower expression in neuronal tissues. The transgenic expression is predominantly only neural, indicated by the black arrows, and in a subset of cells in the pituitary, kidney, lung and adrenal gland. C) Quantitative RT-PCR data for *Cdkn1c *at E13.5 for 5D3 (single copy line) and 5A4 (two copy line) transgenic embryos and 10–15 (*Cdkn1c-βgeo*) at E12.5. *Cdkn1c *levels were normalised against three reference genes: *Gapdh*, *Actin *and *18S *RNA. *Cdkn1c *was at 1.36-fold wild type levels in the single copy line and 1.75-fold endogenous in the two copy line. *Cdkn1c *levels were not significantly raised in the control line, 10–15 (*Cdkn1c-βgeo*). D) Quantitative RT-PCR data for *Cdkn1c *at E12.5 for 5D3 (single copy line) showing the difference in expression between head, where the transgene is predominantly expressed, and the body, where the transgene is only expressed in a small subset of tissues. This is consistent with copy number dependent expression of *Cdkn1c *from the transgene in a subset of tissues.

Following on from our previous work on studying the mechanism of imprinting of *Cdkn1c *[43,44], we investigated the consequences of over expressing *Cdkn1c in vivo *on development. We generated transgenic mice carrying an 85 kb bacterial artificial chromosome (BAC) spanning the *Cdkn1c *locus [44] (Figure 1A). When ES cell lines with low copy number insertions of this transgene (1–4) were aggregated with MF1 morulae, there was a high incidence of perinatal loss (Table 1). No live born chimeras were obtained for high copy lines (Table 1). When surviving chimeras from four independent lines were crossed to out bred MF1 females, transgenic offspring were detected at the expected frequency (Table 2). When these same chimeras were crossed with 129/Sv females, transgenics were recovered at low frequency in a single copy line (1/33) and not at all in three other lines containing 2–4 copies of the transgene (Table 2). We examined litters at earlier time points and dying transgenic embryos were observed at varying stages, some as early as E10.5 (Line 5B6, four copies).

**Table 1:** Contribution of transgenic ES cells to coat colour and germ line transmission in chimeras.

**Line**	**Copy no**.	**Chimeras/total number born**	**% 129/Sv Coat colour**	**% Transmission 129/Sv coat colour**
**5D3**	1	11/11 (5 d.e. pups died)	100	100
			100	100
			100	100
			100	100
			100	100
			100	100
**5C2**	1	10/10 (9 d.e died)	80	12.5
**6C1**	1	2/2 (one d.e. died)	100	100
**5A4**	2	6/6	100	100
			100	100
			100	100
			100	100
			30	100
			0	n.t.
**5A2**	2	7/8 (4 d.e. died)	100	100
			100	100
			100	N.t.
			30	n.t
**5B6**	4	4/9 (2 d.e died)	60	100%
			40 (female)	n.t.
**5A6**	4–8	0/8	-	-
**6A3**	4–8	0/9	-	-
**5A3**	4–8	0/5	-	-
**6A4**	4–8	0/0	-	-

129/Sv ES were aggregated with MF1 morula to generate chimaeric animals containing both ES cells (agouti) and the host MF1 cells (albino). The contribution of ES to the adult chimera was estimated by the percentage of agouti in the coat colour and found to decrease with increasing copy number. Additionally, 50% (21/40) of the pups with dark eyes (d.e.), indicating a strong contribution of ES cells, were born dead or die shortly after birth.

n.t. = not tested.

**Table 2:** Embryonic lethality in transgenic animals on pure a 129/Sv background.

**Line (copy number)**	**MF1 female (Transgenic/Total number)**	**129/Sv female (Transgenic/Total number)**
**5D3/129 (1)**	13/23	1/35*
**Generation 3 5D3**	n.t.	1/33**
**5A4/129 (2)**	3/9	0/16
**5A2/129 (2)**	2/5	0/10
**5B6/129 (4)**	6/10	0/20
**10–15/129 (3)**	N.d.	48/109***

The transgene was present at the expected frequency (50% of the adult offspring, *P *= 0.5) when chimeras were mated with MF1 females but there was a significant reduction in survival of transgenics for single copy line 5D3 (number of transgenic animals/total number genotyped = 1/35, *P = 0.0001) and no survivors for the other lines when chimaeras were crossed with inbred 129/Sv females. F1 males from line 5D3 (50%:50%; MF1 × 129/Sv) were backcrossed onto 129/Sv for three generations (G3). At which point significant lethality was again noted (1/33 transgenics from eight litters, **P = 0.06). The modified transgene (10–15) bred six generations into the 129/Sv background showed no significant difference in survival (***P = 0.20).

We have previously shown that the transgenic *Cdkn1c *transcript is expressed only in a subset of tissues from this 85 kb BAC at E13.5 by *in situ *hybridisation [44]. Immunohistochemistry with a Cdkn1c-specific antibody showed a similarly restricted distribution of the Cdkn1c protein at E12.5, mainly to neural tissues (Figure 1B). Real Time RT-PCR measurements of the *Cdkn1c *transcript revealed an increase in the overall expression of 1.36-fold in whole embryos carrying a single copy of the BAC transgene (line 5D3) and 1.75-fold in embryos carrying two copies (line 5A4) at E13.5 (Figure 1C). This reflects the restricted expression from the BAC which we confirmed in E12.5 embryos by comparing the levels of expression in the head versus the body in the single copy line (Figure 1D). This is consistent with expression from the single-copy transgene at levels similar to the endogenous gene in neural tissues where we know the transgenic *Cdkn1c *to be expressed. We were not able to detect over expression of *Cdkn1c *in the body. We know from our *in situ *data, immunohistochemistry and a reporter transgene [44] that *Cdkn1c *is expression from the BAC in only a subset of the non-neuronal tissues where endogenous *Cdkn1c *is expressed such as the lung endothelium and the developing kidney tubules. The endogenous *Cdkn1c *is expressed most strongly in skeletal muscle and cartilage, where we do not see expression from our transgene. This very strong expression maybe masking the more subtle changes in tissues where there is a relatively lower level expression of *Cdkn1c*. These data are consistent with copy-number dependent expression of *Cdkn1c *from the BAC in a subset of tissues.

### Growth is exquisitely sensitive to the dosage of *Cdkn1c*

**Figure 2:**
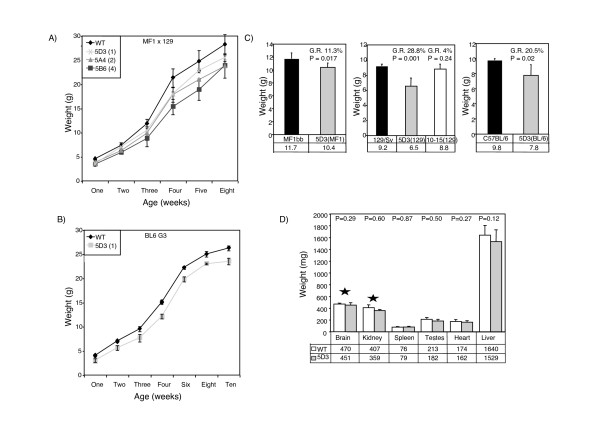
**Postnatal growth retardation in mice carrying *Cdkn1c *transgene**. A) Post natal growth curves of males from one to eight weeks for lines 5D3 (one copy, n = 2), 5A4 (two copy, n = 3) and 5B6 (four copies, n = 2) and their wild type littermates (n = 9). Mice carrying the transgene are significantly smaller in all lines (Statistical significance using Student's *t*-test *P *< 0.017 for all lines). All weights were obtained from F1 mice born from a cross between a chimaeric male founder (129/Sv) and a MF1 female. B) Post natal weight data from one to ten weeks of males for the single copy line, 5D3, bred onto the C57/BL6 background for three generation showing persistence of growth retardation after weaning (one copy, Tg n = 9, WT n = 8). C) Weights at 3 weeks of male mice from line 5D3 (one copy, Tg n = 4, WT n = 9) on a 50%:50%; 129/Sv × MF1 background labelled as 5D3/MF1) compared with a 75%:25% background labelled as 5D3/129 (Tg n = 6, WT n = 8). Interaction with a genetic factor within the 129/Sv background results in a more severe growth retardation, increasing from 11.3% to 28.8% (Statistically significant change using Student's *t*-test. *P *= 0.014). On a C57BL/6 background, the growth retardation is intermediate at 20.5% (Tg n = 9, WT n = 8). No significant growth retardation is observed in transgenic line 10–15 on a 129/Sv background (Tg n = 8, WT n = 4, *P *= 0.24). This line carries three copies of the modified BAC where transgenic *Cdkn1c *is not functional demonstrating that growth retardation is likely due to excess *Cdkn1c*. C) Comparison of organ weights in wild type and 5D3 transgenic adult males at 8–10 weeks. Growth retardation is not restricted to organs in which there was excess embryonic *Cdkn1c *WT n = 9 and Tg n = 6). *denotes tissues that were exposed to excess *Cdkn1c *during embryogenesis.

The transgenic offspring from the cross between the chimeric males and the MF1 females were noticeably smaller than their wild type littermates. The degree of growth retardation increased with increasing copy number of the transgene and persisted after weaning (Figure 2A). Persistent growth retardation was also observed when the single copy transgene was bred onto the C57/BL6 background for three generation (Fig 2B).

The degree of growth retardation also varied with strain background. Transgenic F1 males (50%:50%; 129/Sv:MF1) from a single copy line, 5D3, were 11.3% smaller at three weeks but when these males were crossed with 129/Sv females, the transgenic F2 male offspring (75%:25%; 129/Sv:MF1) were 29% smaller than their non-transgenic litter mates (Figure 2C, two tail student t-Test: *P *= 0.017). Generation 3 C57B/L6 males showed an intermediate phenotype of 20% growth retardation at this time point (Figure 2C, *P *= 0.02). In contrast the lethality associated with an increasing contribution of the 129/Sv genetic background, no evidence of lethality was associated with the presence of a single copy of the transgene on the C57BL/6 background (data not shown).

We examined the age at which males acquired sexual maturity as judged by the timing of their first mating. Despite their small size, transgenic male adult mice reached sexual maturity at the same time as wild type littermates and were fertile (Table 3). The majority of females also reached sexual maturity at a similar age to their wild type littermates but there was one female that had a litter late and two that did not have litters during the time of observation (Table 3). Those that had litters were able to nest and feed their offspring. It should be noted that there is variable fertility in the rare surviving females that are deficient in *Cdkn1c *[45]. The *Cdkn1c*-deficient mice also show a severe postnatal growth defect. This is consistent with a defect in pituitary development. It is possible that, given the expression of *Cdkn1c *from our transgene in the pituitary, further studies on transgenics animals with a higher 129/Sv genetic contribution might reveal an endocrine defect in adults and this is under investigation.

**Table 3:** Excess Cdkn1c does not affect sexual maturity.

	**Age at 1^st ^plug (days)**		**Age at 1^st ^litter (days)**	
	WT males	5D3 male	WT female	5D3 female
	43	39	54	58
	38	37	56	69
	39	38	58	57
	37	38	56	55
	46		56	56
	37		55	57

**Average (s.d.)**	40 (3.69)	38 (0.82)	56 (1.33)	59 (5.16)

There was no significance difference in time to first plug between transgenic males and wild type littermates (*P *= 0.33). Transgenic females had their litters slightly later than wild type females. Although this difference did not show up as significant (*P *= 0.22), there was a large variation in timing within the transgenic females from 56 to 69 days. Furthermore, two females in this study never became pregnant.

Cdkn1c is a cell cycle inhibitor that acts intrinsically to limit cell proliferation. We would expect that the tissues in which there was excess *Cdkn1c *would be disproportionately smaller than those without. We examined the weights of organs with no excess *Cdkn1c *(heart, spleen, testes and liver) and those in which there had been excess embryonic *Cdkn1c *(kidney and brain, Figure 2D) in eight-week old males. There were no significant difference in comparison to body weight (P = 0.12–0.87) between wild type and transgenic males. Growth retardation induced by the presence of a single copy BAC was not restricted to the tissues in which the transgenic *Cdkn1c *was expressed.

**Figure 3:**
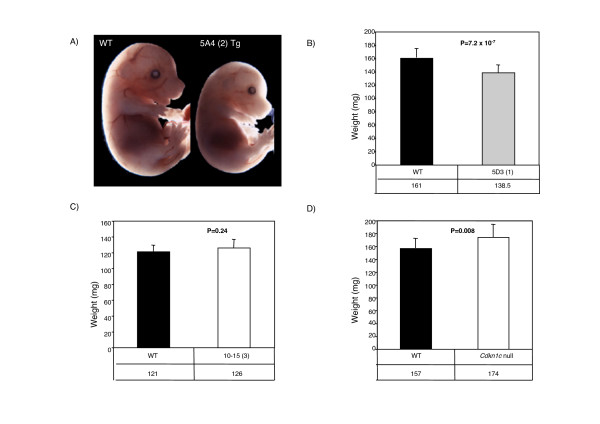
**Embryonic growth is exquisitely sensitive to *Cdkn1c *levels**. A) Image of a wild type and a transgenic embryo from line 5A4 (two copies) at E16.5 shows demonstrable growth retardation. The embryos appear normally proportioned. B) A comparison of wild type and transgenic embryo weights at E13.5 for line 5D3 (single copy unmodified BAC, Tg n = 22, WT n = 21). These embryos are from 129/Sv females mated with transgenic males maintained on an MF1 background. Transgenic embryos weigh significantly less than wild type embryos (86%, *P *= 7.2 × 10^-7^). C) A comparison of wild type and transgenic weight at E13.5 of embryos for line 10–15 (three copy BAC with an inactive transgenic *Cdkn1c *locus and no excess *Cdkn1c*, Tg n = 30, WT n = 22). The line was maintained on a 129/Sv background. Transgenic embryos are not significantly different in weight to wild type embryos (104%, *P *= 0.24). D) A comparison of wild type and transgenic weights at E13.5 of embryos inheriting a targeted *Cdkn1c *allele maternally (null for *Cdkn1c*, Tg n = 19, WT n = 16). The line was maintained on a 129/Sv background. Transgenic embryos weigh significantly more than wild type embryos (111%, *P *= 0.008).

The transgenic embryos were small but similar in appearance to wild type embryos (Figure 3A) in all four lines. At E13.5, transgenic embryos from line 5D3 (single copy) on a mixed MF1:129/Sv background are 14% smaller than wild type (Figure 3B; *P *= 7.2 × 10^-7^). This phenotype is comparable to that reported for the *KvDMR1 *deletion and the 800 kb YAC where several additional genes are also over expressed [25,42]. In contrast, embryos null for *Cdkn1c *were 11% heavier wild type embryos at E13.5 (Figure 3D, *P *= 0.008).

### Phenotype is due to excess *Cdkn1c*

The transgene spans two other genes, *Phlda2 *(also known as *Ipl *or *Tssc3*) [46] and *Slc22a18 *(also known as *Impt1 *or *Tssc5*) [47]. The growth retardation and embryonic lethality could be a consequence of over expression of any one of these genes. To test this possibility, we examined the transgenic line, 10–15 (*Cdkn1c-βgeo*). This line contains three copies of the same BAC but the *Cdkn1c *gene on the BAC has been inactivated by the insertion of a reporter gene (Figure 1A) [44]. In these mice, *Cdkn1c *is not over expressed in the embryo (Figure 1C). *Phlda2 *and *Slc22a18*, which are predominantly expressed in extra embryonic tissues [45,47], are highly over expressed in the placenta [48] but not detectable in the embryo at E13.5 (data not shown).

No embryonic lethality was observed when we first generated this line or when we backcrossed the mice for six generations onto the 129/Sv background (Table 2). This contrasts with almost complete loss of transgenic animals (1/34 transgenic/wild type) after crossing 5D3 F1 males (50:50; 129/Sv × MF1) onto the 129/Sv background for three generations (>93% 129/Sv). There was no significant difference in weights between males from line 10–15 (*Cdkn1c-βgeo*) and wild type littermates at three weeks (Figure 2B, *P *= 0.24) and embryos from line 10–15 (*Cdkn1c-βgeo*) are not significantly different to their wild type counter parts (*P *= 0.24) at E13.5 (Figure 3C). This line was generated by pronuclear injection and it was possible that we selected against any transgenic lines showing an adverse phenotype. Therefore, we also examined embryos at E13.5 after a second round of pronuclear injection. Seven embryos representing independent integration events were identified. No significant difference in embryonic size was noted (data not shown).

Placental insufficiency can also cause embryonic growth retardation in mice as illustrated by the placental specific *Igf2 *knock out [49]. Both the unmodified and modified BAC transgenic lines have a placental defect due to excess *Phlda2 *and/or *Slc22a18 *([48] and our unpublished data). In the absence of over expression of *Cdkn1c *in line 10–15 (*Cdkn1c-βgeo*), this placental defect leads to a slow down in embryonic growth detectable at E16.5 [48]. In the lines with excess *Cdkn1c*, we observe embryonic growth retardation three days earlier, at E13.5. Our control line 10–15 (*Cdkn1c-βgeo*) embryos are of normal weight at E13.5 (Figure 3C). Therefore, this earlier defect in embryonic growth is due to excess *Cdkn1c *and not due to the placental insufficiency shared by both lines. Furthermore, a key characteristic of IUGR due to placental insufficiency in mice is postnatal "catch up" growth [49]. Our mice do not recover their growth potential after birth (Figure 2A). Even at one year, they are noticeably smaller than their wild type siblings (data not shown).

We can conclude that excess *Cdkn1c *in the embryo causes sustained growth retardation and, on the 129/Sv genetic background, also causes embryonic lethality.

### *Igf1 *expression is reduced as a consequence of increased *Cdkn1c *levels

**Figure 4:**
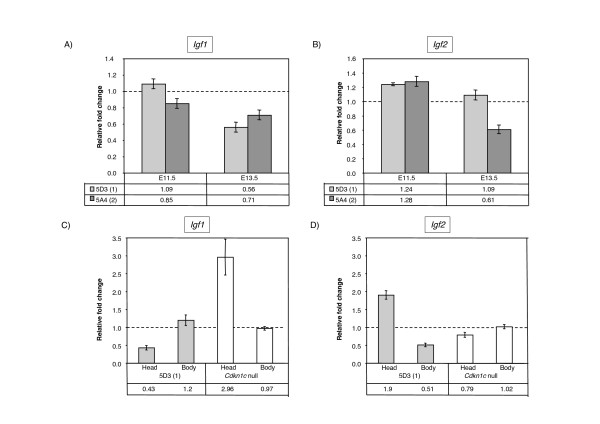
**Quantitative analysis of growth factor expression levels in response to excess embryonic *Cdkn1c***. A) Quantitative RT-PCR data for *Igf1 *on whole embryos from lines 5D3 and 5A4. At E11.5, there is a slight reduction in *Igf1 *in the two-copy line. At E13.5, there is a reduction in *Igf1 *levels in both lines. B) Quantitative RT-PCR data for *Igf2 *on whole embryos from lines 5D3 and 5A4. At E11.5, there is a slight increase in *Igf2 *in both lines while at E13.5, there is a significant decrease in *Igf2 *in the two-copy line. C) Comparison of expression of *Igf1 *at E12.5 in the single copy line, 5D3, head compared with body and whole *Cdkn1c *null embryos. In line 5D3, *Igf1 *levels are 60% less than wild type levels in the head where transgenic *Cdkn1c *is predominantly expressed but relatively unaffected in the body. In null embryos, *Igf1 *levels are raised by three-fold in the head. D) Comparison of expression of *Igf2 *at E12.5. In line 5D3, *Igf2 *levels are raised by two-fold in the head but 50% wild type levels in the body. In null embryos, *Igf2 *levels are similar to wild type.

Cdkn1c acts intrinsically to regulate cell proliferation and, in some tissues, also directs differentiation. However, growth retardation was not limited to the tissues in which there was an embryonic excess Cdkn1c. One possible explanation was that excess Cdkn1c altered the expression level of one of the key embryonic growth regulatory genes which would then have an extrinsic effect on growth. We used quantitative Real Time RT-PCR to measure the transcript levels of several genes known to regulate embryonic growth. At E11.5 we identified a reduction in *Igf1 *in our two-copy line but not in our single-copy line. *Igf1 *was reduced in both the single-copy and the two-copy transgenic lines at E13.5 when there was obvious growth retardation (Figure 4A). *Igf2 *levels were slightly raised at E11.5 in both lines and reduced at E13.5 only in the two-copy line (Figure 4B).

In the single copy line, we only detected changes in *Igf1 *at E13.5, when the growth retardation phenotype was well established. In order to detect more subtle changes in gene expression at an earlier time point in this line, we examined *Igf1 *and *Igf2 *levels in RNA prepared separately from the head and in the body of E12.5 embryos. *Cdkn1c *expression from our transgene was predominantly in neural tissues and we reasoned that we would be more likely to detect a change in expression in material where this tissue predominates. When we examined the transgenic embryos, we found that the level of *Igf1 *in the head was dramatically reduced to 40% of the wild type level (Figure 4C). In the body, where we have minimal over expression of Cdkn1c, *Igf1 *levels were not significantly changed from normal. The central nervous system is a major site for expression of *Igf1 *[50,51]. Consistent with this observation, we found that, in wild type embryos, there was 9.5-fold more *Igf1 *RNA in the head than in the body at E12.5 (data not shown). *Igf2 *levels were raised in the head by two-fold and reduced in the body by 50%. Since the body (mesodermal tissues) is the primary source of *Igf2 *[2], the overall change in expression in the transgenic embryos was not significant.

To provide further data to support a role for Igf1 in the growth phenotype, we examined *Igf1 *and *Igf2 *levels in RNA prepared from the heads and bodies of embryos null for *Cdkn1c *at E12.5. We again found changes in the level of expression of *Igf1 *with a three-fold increase in *Igf1 *expression levels in the head and difference in expression in the body (Figure 4C). *Igf2 *levels were relatively unchanged in both samples (Figure 4D).

We have found an inverse correlation between the level of expression of *Cdkn1c *and the level of expression of the potent growth factor *Igf1*. These data are consistent with a role for this growth factor in our growth phenotype. *Igf1 *levels are linked, either directly or indirectly, to level of expression of *Cdkn1c *and growth is either retarded or enhanced as a consequence.

We have shown that Cdkn1c acts as a true rheostat for embryonic growth, with loss of expression after gene deletion leading to overgrowth and over expression of Cdkn1c causing growth retardation.

## Discussion

### Embryonic growth is exquisitely sensitive to the dosage of *Cdkn1c*

Excess expression of *Cdkn1c *from a single copy BAC transgene resulted in decreased growth of embryos from at least E13.5 with no catch-up growth after birth. Furthermore, as the level of excess *Cdkn1c *increased, the size of the animals decreased. Initial reports for the phenotype of *Cdkn1c *null mice did not reveal a clear affect on growth at birth [31,32]. However, we found that loss of *Cdkn1c *was associated with embryonic overgrowth earlier in development, at E13.5. Therefore, *Cdkn1c *acts as a true rheostat for embryonic growth.

### *Cdkn1c *is the most important regulator of embryonic growth within the IC2 domain

**Figure 5:**
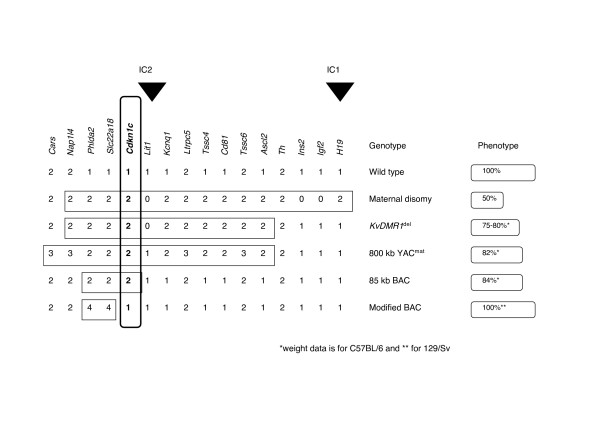
**Cdkn1c is the key regulator of embryonic growth within the IC2 imprinted domain on mouse distal chromosome 7**. Summary of the reported genotypes and phenotypes associated with the imprinted genes on mouse distal chromosome 7. Black arrowheads indicate the positions of the known IC for the two domains. The number of expressed gene copies present in each genotype is shown with the top row being the normal, wild type state. The reported weight phenotypes are for maternal disomy distal chromosome 7 [22], paternal inheritance of the *KvDMR1 *deletion [25], maternal inheritance of an 800 kb YAC [42] and data from this paper. The strain backgrounds are C57BL/6 where indicated.

Studies on mono-parental embryos and embryos with paternal or maternal disomy of specific chromosomal regions revealed a key role for imprinted genes in regulating embryonic growth. These studies invariably involved both loss of expression and excess expression of multiple genes and the identification of the target for imprinting of the locus is difficult. Embryos with maternal disomy of mouse distal chromosome 7 showed embryonic and extra embryonic growth retardation and embryonic lethality [22] with loss of expression of *Igf2 *and *Ins2 *and excess expression of a number of genes presumed to include *Cdkn1c*, *Ascl2 *and *Phlda2 *(summarised in Figure 5). Paternal inheritance of a targeted deletion of the *KvDMR1*, which resulted in loss of imprinting of only the IC2 domain, resulted in a slightly less severe growth retardation [25] similar to maternal inheritance of the 800 kb YAC spanning all the genes within this domain [42]. Our unmodified BAC, which spans only three of these genes, also caused embryonic growth retardation. The modified BAC, which drives excess *Phlda2 *and *Slc22a18 *but not excess *Cdkn1c*, did not. Therefore, *Cdkn1c *is the only gene in this region that has been shown to have a major role in regulating embryonic growth.

### Embryonic lethality in embryos with maternal disomy of the distal 7 domain may be due to excess *Cdkn1c*

There are differences in survival between embryos with maternal disomy of distal chromosome 7 and those with paternal inheritance of the *KvDMR1 *deletion. This could be interpreted to indicate that the embryonic lethality in the mice with maternal disomy of distal 7 was due to loss of *Igf2 *and/or *Ins2*. However, embryonic lethality was not been reported for loss of *Igf2 *[2] or *Ins2 *[52] alone. Although lethality could be due to a combination of loss of *Igf2 *with excess *Cdkn1c*, we have observed embryonic lethality associated with excess *Cdkn1c *and normal *Igf2 *levels on the 129/Sv genetic background.

### Altered expression of *Igf1 *in response to changes in the dosage of *Cdkn1c*

Our data showed that increasing expression levels of *Cdkn1c *were associated with a decrease in the size of the embryo, starting prior to E13.5. As the level of *Cdkn1c *increased, we found a consistent decrease in the transcript level of the potent embryonic growth factor, *Igf1*, which was detectable as early as E11.5 in our two-copy line. Conversely, loss of expression of *Cdkn1c *was associated with a reciprocal phenotype with the null embryos being 11% heavier than wild type embryos at E13.5 with a three-fold increase in *Igf1 *at E12.5. Complete loss of *Igf1 *results in embryonic growth retardation from E13.5 [53,54]. Similar to our 5D3 mice, *Igf1 *heterozygotes are 10–20% smaller at birth and remain this small as adults [54]. Although no significance size difference was reported at E13.5 in the *Igf1 *heterozygotes, genetic modifiers may provide an explanation for this discrepancy since our preliminary data suggest a weaker growth retardation phenotype in the C57BL/6 background on which the *Igf1 *studies have all been performed.

We also observed disturbances in the level of expression of a second key growth factor transcript, *Igf2*. However, this was only in the *Cdkn1c *overexpressing mice and not in the *Cdkn1c *null mice. Also, we have some limited data from genetic crosses with mice over expressing *Igf2 *[55] that do not support a major role for this growth factor in our phenotype. It is therefore probable that reduced expression of *Igf1 *was responsible for growth retardation in the presence of excess *Cdkn1c*. This would also provide an explanation for why growth retardation was not restricted to the tissues in which there was excess *Cdkn1c *since Igf1 acts extrinsically to regulate growth. It will be possible to test this in genetic crosses when mice over expressing *Igf1 *from an endogenous promoter become available.

There are a number of ways in which the cell cycle inhibitor, *Cdkn1c*, could act on *Igf1*. Although we cannot exclude a direct role for *Cdkn1c *in regulating the expression of the *Igf1 *gene, it seems more plausible that the cell cycle inhibitor acts indirectly, for example by reducing the proliferative potential of the cells in which *Igf1 *is expressed or by altering the differentiation program of a particular cell type expressing the growth factor.

While it has been known for some time that imprinting acts directly to regulate embryonic growth via the epigenetic modification of key genes, our data suggests that imprinting can also act indirectly to regulate the levels of non-imprinted embryonic growth factors. This further highlights the complexity and the importance of genomic imprinting in mammalian growth and development.

### Imprinting of *Cdkn1c *comes at a high cost

Loss of expression of this gene is perinatal lethal in mice and linked to BWS in humans [33-37]. We have shown that excess *Cdkn1c *can be equally detrimental to development in mice and there is some evidence for this in humans [56]. Given the increased frequency of intrauterine growth retardation in babies conceived after assisted reproductive technologies conceptions [57-59] and a prevalence of imprinting mutations in these children [60-63], excess *CDKN1C *may be an important factor in IUGR in humans which should be explored further.

### Imprinting of *Cdkn1c *may have played a role in the transition towards increased maternal investment in embryonic growth in placental mammals

The parental conflict hypothesis predicts that over expression of a gene expressed only from the maternal genome should limit embryonic growth. We have presented the first compelling evidence that overexpression of *Cdkn1c *alone causes growth retardation and therefore the parental conflict hypothesis holds true for this gene. Our work may also provide further clues to the evolutionary consequence of imprinting this important gene. Imprinting has for some time been thought to have emerged prior to the divergence of marsupials from eutherians [64-66]. However, unlike *Igf2, Cdkn1c *is not imprinted in marsupials [67]. Therefore, the IC1 domain, containing the potent growth factor gene, *Igf2*, acquired an imprint prior to the divergence of marsupials and eutherians and the IC2 domain, containing *Cdkn1c*, became imprinted after this divergence. It may be significant that one key difference between marsupials and eutherians is the size and maturity of the offspring at birth and this correlates with the role that we have identified for *Cdkn1c *in regulating embryonic growth.

## Conclusion

By using different copy number transgenes and mice carrying a targeted deletion of *Cdkn1c*, we have shown that embryonic growth is exquisitely sensitive to the dosage of *Cdkn1c*. Since *Cdkn1c *encodes the major regulator of embryonic growth within the IC2 imprinted domain, we propose that *Cdkn1c *was the focal point of the selective pressure for imprinting of this domain. *Cdkn1c *is a maternally expressed gene and our findings support the prediction of the parental conflict hypothesis that that the paternal genome will silence genes that have an inhibitory role in embryonic growth. Furthermore, since *Cdkn1c *acquired its imprinted status after marsupials diverged from placental mammals, our data would support a role for the change in dosage of *Cdkn1c *gene in the relative increase size of the embryo in eutherians.

## Methods

### Mice

All animal studies and breeding was approved by the Universities of Cambridge and Cardiff ethical committees and performed under a UK Home Office project licenses. Inbred 129/Sv and C57BL/6 and out bred MF1 mice were maintained at the Gurdon Institute, Cambridge, UK and Cardiff School of Biosciences. Additional 129/Sv mice were purchased from B+K Universal. Transgenic lines carrying the 85 kb bacterial artificial chromosome were described previously [44,68]. Essentially, ES cell lines were made by co-electroporation of the linearised BAC with a linearised *pgk-neomycin *construct and selecting for neomycin positive colonies. Genomic DNA from these colonies was analysed by Southern blotting. Intactness and copy number of the BAC integrations was determined by probing the membrane first with the complete 85 kb BAC (after blocking repeats with total mouse genomic DNA) and then with a single copy probe to *Neuronatin *[69]. Lines carrying 1–8 copies of the BAC were co-cultured with MF1 morulae to generate chimaeric animals. The lines were maintained on a mixed 129/Sv × MF1 background. F1 5D3 (50%:50%; 129/Sv × MF1) denotes animals that have an equal contribution of MF1 and 129/Sv from a cross between a male chimera and a MF1 female, F2 5D3 (75%:25%; 129/Sv × MF1) denotes animals that have a greater contribution of 129/Sv obtained by crossing a F1 5D3 (50%:50%; 129/Sv × MF1) male with a 129/Sv female. Animals were genotyped by PCR using primers that identified the pBeloBAC vector sequence (R85: 5'-attctgcttacacacgatgc-3' and R86: 5'-ttccgcagaggtcaatcc-3'). Generation of line 10–15, which contains the modified BAC transgene with an inactivated *Cdkn1c *gene (*Cdkn1c-βgeo*), was previously described [44]. These mice were maintained on a 129/Sv background as were mice carrying a targeted allele of *Cdkn1c *[31]. All mice were tailed between P7 and P14 and genomic DNA for genotyping was prepared as described [70].

Sexual maturity was determined by daily checking for plugs and the appearance of a litter.

### Weighing studies

Postnatal weights of males were taken at weekly intervals for one to eight weeks. At 8–10 weeks, males were killed by cervical dislocation. Organs were dissected and kept frozen on dry ice before weights were obtained. Embryonic weights were taken 11, 12 or 13 days after a discernable plug, at E11.5, 12.5 or 13.5. Embryos were stored in RNAlater (Sigma) while genotyping data was obtained from yolk sac DNA.

### Statistics

Statistical significance (Probability values) was determined using the Student's *t*-Test (two tailed distribution and two sample unequal variance). The significance in the difference in observed over the expected appearance of a particular genotype was determined using the Chi-squared Test.

### Quantitative RNA analysis

Total RNA was prepared from embryos using TRI Reagent (Sigma). cDNA was prepared using M-MuLV reverse transcriptase (Roche) with random hexamers under the manufacturers conditions. 20 μl cDNA was diluted five-fold in 10 mM Tris.HCl (pH 7.5) and stored at -20°C. For the quantitative PCR, primers were designed to amplify across introns for all the genes using the Primer3 program [71] unless otherwise stated. Quantitative RT-PCR was performed in duplicate on two independent samples and on two separate occasions on diluted RT^+ ^and RT^- ^material prepared for wild type and transgenic embryos using DyNAmo HS SYBR Green qPCR kit (Finnzymes), GRI 96 PCR detection plates and ABsolute QPCR seal (ABgene) and detected using the Chromo 4 Four-Color Real Time detector (MJ Research). Conditions for amplification: 15 minutes at 95°C (to release polymerase) followed by 40 cycles of 30 seconds at 95°C and 1 minute at 63°C (anneal and extension). 1 μl of diluted cDNA was amplified in a total sample volume of 25 μl with 25 pmoles of each primer. Primers were 5'-agagaactgcgcaggagaac-3' and 5'-tctggccgttagcctctaaa-3' for *Cdkn1c*, 5'-ggcattgtggatgagtgttg-3' and 5'-tctcctttgcagcttcgttt-3' for *Igf1*, 5'-gtcgatgttggtgcttctca-3' and 5'-aagcagcactcttccacgat-3' for *Igf2 *and 5'-cgctgagccagtcagtgtag-3' for *18S*. *Gapdh *and *Beta-actin *primers were as previously described [72,73].

### Immunohistochemistry

E12.5 embryos were fixed in pre-cooled Carnoys (60% ethanol, 30% chloroform, and 10% glacial acetic acid) for 25 min at 4°C, then washed three times for 2 min in 96% ethanol, then 100% ethanol for 2 and 7 min, respectively. Embryos were then cleared in 1:1 ethanol/chloroform (V/V) for 10 min and chloroform for 10 min, then 1hr. Embryos were then impregnated in three changes of 56–58°C melting point paraffin wax (Sigma-Aldrich) for 30 min each at 65°C and embedded. 10 μm sections were dewaxed in xylene and rehydrated through a series of graded ethanol. Washes in PBS with 0.02% Tween 20 were carried out between each subsequent step. Endogenous peroxidases were quenched by incubation in 3% hydrogen peroxide in water for 5 min, then 0.5% hydrogen peroxide in methanol for 1 hr. Sections were blocked in 2.5% horse blocking serum (RTU VECTASTAIN universal quick kit, Vector Labs) for 1 hr. Goat anti-CDKN1C primary antibody M-20 (SC-1039, Santa Cruz) was diluted 1/50 in 0.04% horse blocking serum and incubated at 4°C overnight. Primary antibody was removed and secondary antibody and streptavidin/peroxidase steps were carried out as per manufacturers instructions (RTU VECTASTAIN universal quick kit, Vector Labs). Sigma Fast DAB substrate system was used to visualise staining. Sections were counterstained with Gills III hematoxylin for 1 min then destained for 1 min with acid/alcohol solution (0.5% HCl, 70% ethanol). Sections were dehydrated through a series of graded ethanol and washed in xylene before mounting with DPX mounting medium.

## Abbreviations

BAC Bacterial Artificial Chromosome

BWS Beckwith-Weidemann Syndrome

β-*geo *β-*galactosidase-neomycin*

IC Imprinting Centre

PCR Polymerase Chain Reaction

RT Reverse transcriptase

YAC Yeast Artificial Chromosome

## Authors' contributions

RMJ was the major contributor to conception and design, acquisition of data, analysis and interpretation of data and generated the *Cdkn1c *transgenic lines in the study and drafted the manuscript. MAS contributed to conception and design. SCB generated the control transgenic line and participated in the initial characterisations. SCA was involved in the acquisition of data, analysis and interpretation of data. MDW designed and implemented qRT-PCR studies while SJT dissected animals for data and managed the transgenic colonies.

All authors read and approved the final manuscript.
